# Clinical Science in the Chemical Senses: Mechanisms, Perception, Behaviors, and Disorders

**DOI:** 10.1093/chemse/bjaa054

**Published:** 2020-11-06

The prevalence of chemosensory loss associated with the COVID-19 pandemic, as well as the growth of patient-focused efforts around the world have significantly raised public and health provider awareness of chemosensory disorders, their consequences for health and quality of life, and the importance of the chemical senses for our daily lives. This increased interest in smell, taste, chemesthesis, and flavor perception offers a unique opportunity for clinical and pre-clinical researchers to advance our understanding of the chemical senses, their normal contributions to perception and behavior, and the mechanisms and potential treatments for chemosensory disorders. This special issue will highlight original research and informed perspectives in these areas.

To be included in this special issue, submissions should make a new contribution to the empirical and/or theoretical understanding of human chemosensory function or dysfunction, including normal chemosensory perception, chemosensory disorders, and diagnosis. We welcome—but do not exclusively focus on—manuscripts that present work related to COVID-19-associated chemosensory dysfunction. Submissions may consist of original research (observational, experimental, and clinical trial studies) or reviews (systematic or narrative). If you wish to submit a review, opinion paper, or case study, please contact one of the listed editors below prior to submitting. We also welcome chemosensory pre-clinical research that uses non-human animal models or in vitro approaches in this special issue as long as potential clinical applications are clearly apparent. Papers should meet the high scientific and ethical standards of *Chemical Senses* and will be subject to a normal review process. A cover letter should summarize the main scientific contributions and how the paper fits with the focus of the special issue. Manuscripts thought to be outside the scope of this special issue can still be considered for publication in *Chemical Senses* as a stand-alone paper through the normal review process.

**Submission deadline for this special issue is 31 January 2021**. If revisions are invited by the editors, these will typically be expected within 2 months of receipt of the editorial decision letter and accompanying reviews to facilitate a timely publication process. Submitted manuscripts will be published upon acceptance, and assembled into an online special issue, thereby providing no delay compared to the regular review process.

*Chemical Senses* publishes original research, methodological advances, and review papers on all aspects of chemoreception in both humans and animals.

Submission site: https://mc.manuscriptcentral.com/chemsens

Submission guidelines: https://academic.oup.com/chemse/pages/instructions_for_authors

Editors for this special issue:

Dr. Johan N. Lundström, Karolinska Institutet, Sweden; johan.lundstrom@ki.se

Dr. Sanne Boesveldt, Wageningen University, the Netherlands; sanne.boesveldt@wur.nl

